# Analysis of Xinjiang asphaltenes using high precision spectroscopy

**DOI:** 10.1039/d0ra07278h

**Published:** 2020-10-27

**Authors:** Xiong Qiyong, Kiyingi Wyclif, Pan Jingjun, Ruiying Xiong, Weibing Deng, Shiling Zhang, Jixiang Guo, Yuqi Yang

**Affiliations:** PetroChina Karamay Petrochemical Co. Karamay Xinjiang 834000 China; Unconventional Petroleum Research Institute, China University of Petroleum Beijing 102249 China guojx003@163.com; State Key Laboratory of Heavy Oil Processing at Karamay, China University of Petroleum-Beijing at Karamay Karamay 834000 China

## Abstract

Asphaltenes are known for causing flow assurance problems in numerous oil fields. In this study we present a comparative spectroscopic analysis of Xinjiang heavy oil asphaltenes as part of ongoing research for an environmentally friendly and cheap chemical inhibitor. The goal is to predict the internal morphology of these asphaltenes through comparative analysis using high precision spectroscopy. Fourier transform infrared spectroscopy (FTIR), proton-nuclear magnetic resonance (H-NMR) and electrospray ionization Fourier transform ion cyclotron resonance combined with mass spectroscopy were used in this analysis. Several studies have demonstrated the enormous potential of these techniques to characterize hydrocarbons. Here we comparatively apply these techniques to characterize Xinjiang asphaltenes with reference to earlier imaging studies with atomic force and scanning tunneling microscopy to assign a structure to these asphaltenes. Results revealed the nature of the asphaltenes to be polycyclic, aromatic with both heteroatomic and metallic content. Thirteen basic and eleven non-basic/acidic nitrogen compounds fused within the aromatic network were identified. The mass distribution is in the range between 100–800 Da. H-NMR revealed various structural parameters (aromaticity and degree of unsaturation) and together with FTIR various functional groups were identified that include: ethers, sulphides, amides and sulfoxides. The predicted structures are consistent with the “island” and “aryl linked core” models.

## Abstract 2

Asphaltenes are known for causing flow assurance problems in numerous oil fields.

## Graphical abstract

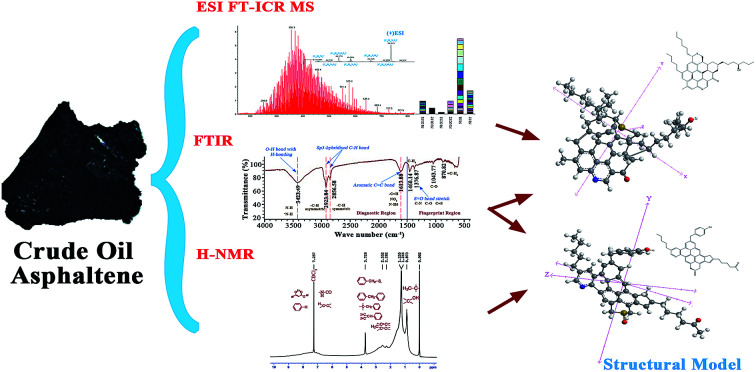

## Introduction

Among the problems during the production of heavy oil in Eastern China is the precipitation of asphaltenes. Therefore, their mitigation and control is of vital importance. Among the most used mitigation measures is the use of chemical inhibitors or dispersants to alleviate asphaltene related problems. During formulation of remediation chemicals; it is imperative to know the structural chemistry of asphaltenes, yet their complexity makes this challenging.^1^ The asphaltenes used in this research were extracted from Xinjiang crude oil. The oil field is a carbonate reservoir located in the northwest of china and is characterized by shallow burial and low temperatures. The oil is categorized as heavy due to low H/C ratio and high viscosity.^2^ For analysis purposes the oil can be separated into four fractions: saturates, aromatics, resins and asphaltenes. Separation/extraction of these components simplifies oil characterization as each fraction can be independently analysed.^3,4^ Saturates are aliphatic, non-polar, branched or linear including cycloalkanes. Aromatics have one or more benzene rings. On the other hand, resins and asphaltenes both have high molecular weights and contain heteroatoms. The difference lies in their solubility in non-polar solvents. Whereas resins are soluble in pentane and in higher alkane solvents, asphaltenes are insoluble in the later but soluble in benzene and toluene.^3–6^

Quantitative analysis for SARA fractions shown in Table 1 substantiates the asphaltene problem in this crude oil. According to oil stability models, this disproportionality between asphaltenes and resins risks the stability of the oil that consequently results into aggregation and deposition of asphaltene blockages causing flow assurance problems, increased work overs and pipe replacements.^7,8^

**Table 1:** SARA fractions in Xinjiang crude oil

Oil	Saturates%	Aromatics%	Resins%	Asphaltenes%
XJ-1	11.70	41.86	18.16	28.28
XJ-2	17.69	27.92	10.45	43.94
XJ-3	13.13	28.12	8.93	49.82

Indeed, over the past two decades a number of spectroscopic and microscopic studies have been used to deduce the structural morphology of asphaltenes.^9–11^ The most prominent breakthrough was in 2015 when a team at IBM Zurich were able to provide for the first-time microscopic images for 100 asphaltenes from both coal and crude oil revealing the molecular structures of asphaltenes.^12^ This was achieved through use of a custom made experimental setup that combined scanning tunnelling (STM) and atomic force (AFM) microscopy. These breakthroughs have to a great extent provided answers as well as providing evidence to earlier debates on molecular weight and structural models of asphaltenes.^13–16^ It's now generally accepted that “island” or “aryl linked core” models are more representative of asphaltenes whereas the archipelago model has been either redefined or has been neglected entirely.^13^ With this new information coming to light; in this paper we combine this microscopy information with results obtained using renown spectroscopic methods to characterize and assign chemical structures to these asphaltenes. Various studies in the past have shown that these methods can be used to effectively characterize asphaltenes although did not answer the questions on the type of model.^13,14^

### ESI FT ICR MS mass spectrometry

This is an ionization technique that involves electrospray ionization coupled with Fourier transform-ion cyclotron mass spectroscopy (ESI FT-ICR MS).^17–22^ Xinjiang oil contains considerable amounts of heteroatoms,^2^ thus their analysis is warranted. The ESI FT-ICR MS mass spectra provide ultra-high resolution and quality accuracy, enabling the precise determination of mass distributions of heteroatoms for both basic and non-basic nitrogen compounds (BNC and NBC) in the asphaltenes as well as approximate asphaltene molecular weight. This is because these compounds respectively can be ionized in both the positive and negative. Electrospray ionization modes.^20^ Moreover, the carbon number and degree of unsaturation can as well be deduced.^23^ The degree of unsaturation/condensation is represented by the double bond equivalent index (DBE), eqn (1).^9,24^1
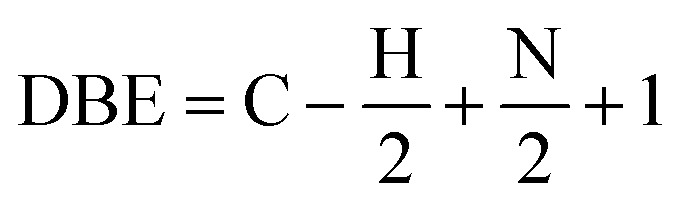


The lower the DBE, the more aliphatic the molecule and degree of saturation. Whereas the higher the DBE, the high the degree of aromaticity and unsaturation/condensation.

### Nuclear magnetic resonance (^1^H-NMR)

This method is key in identifying chemical environments through analysis of the chemical shifts in the spectrum that are vital in determination of asphaltenes' structural parameters like aromaticity, degree of branching, aromaticity and naphthenic ring numbers among others.^25^

Aromaticity, *f*_A_, of asphaltenes can be determined using the modified Brown and Lander method that utilizes the intensities of the resonated aromatic (H_A_) and alkyl hydrogen atoms (H_α_, H_β_, H_γ_) using eqn (2).^26,27^2
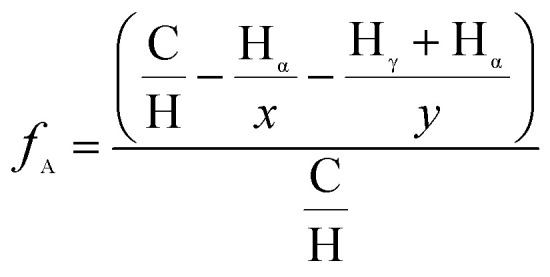
where C and H are carbon and hydrogen values obtained from elemental analysis, *x* and *y* represent average number of hydrogens per alpha-alkyl and gamma-alkyl, respectively.

Apart from, aromaticity, the average carbon number per alkyl substituent, *n*, is obtained from the hydrogen spectrum and calculated as per eqn (3).3
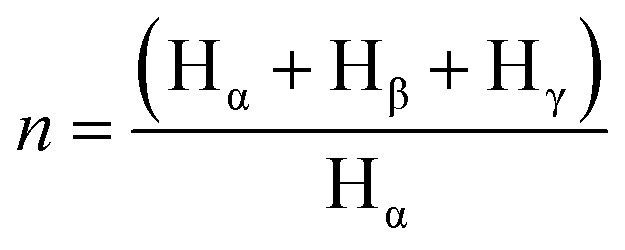


Various methods for calculations are used to determine the parameters and reference is given to the works by D. R. Clutter and his team.^28^

### Fourier transform infrared spectroscopy

This spectroscopy is based on the principle that bonds between molecules act just like springs, thus they can absorb energy and undergo vibration or stretching. The energy, *E*, absorbed is directly proportional to the wave number, *ν*, of vibration (eqn (4)) and it is inversely proportional to the wavelength, *λ*, (Beer's law).^29,30^4
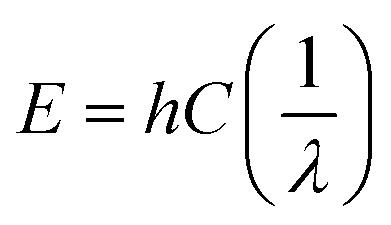
where *h*-Planck's constant (kg m^2^ s^−1^ or J s^−1^) and *C*-velocity of light (m s^−1^).

And from Hooke's law, wavenumber can be calculated as;5
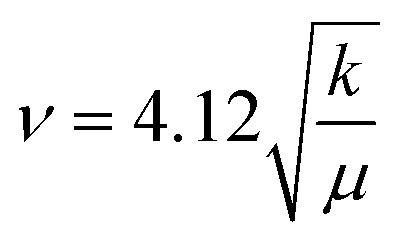
where *k* is bond strength (dynes cm^−1^) and *μ* is the reduced mass of the bonding atoms.

As a result, this technique can determine the functional groups in the asphaltenes that together with the NMR spectroscopy can give a detailed insight into the asphaltenes molecular structure and assembly units.^31,32^

This study focuses on the use these techniques in unison to provide a more detailed comparative understanding of the internal structure of the asphaltenes to form logical conclusions.

## Results and discussion

### Elemental analysis

Non-metallic and metallic elemental composition in the asphaltenes is presented in Table 2. This experimental data is key in determining structural properties of the asphaltenes especially the hydrogen and carbon quantities.

**Table 2:** Analysis of asphaltene metallic and non-metallic elements

Non-metals								Metals	
Asphaltene	C%	H%	S%	N%	O%	(NSO) %	H/C	*V* (μg g^−1^)	Ni (μg g^−1^)
XJ-1	74.29	6.00	3.15	1.08	3.27	7.50	0.9692	618	92
XJ-2	77.67	6.72	3.38	1.17	2.75	7.31	1.0382	795	106
XJ-3	74.87	6.34	3.38	1.26	3.13	7.77	1.0162	852	109
Mean	75.61	6.35	3.30	1.17	3.05	7.53	1.0078	755	307

It can be seen that the asphaltenes are characterized by a lower nitrogen content as compared to oxygen and sulphur heteroatoms, Table 2. It was also observed that the variations in non-metallic elements of the three samples is significantly small.

The low nickel to vanadium ratio is indicative of the origin of the crude oil from which the asphaltenes were extracted (carbonate reservoir). They make up the smallest portion of the asphaltenes and for this reason we considered their contribution to structural assignments to be inconsequential.

### ESI FT-ICR MS analysis of XJ-1 asphaltenes

**Fig. 1:**
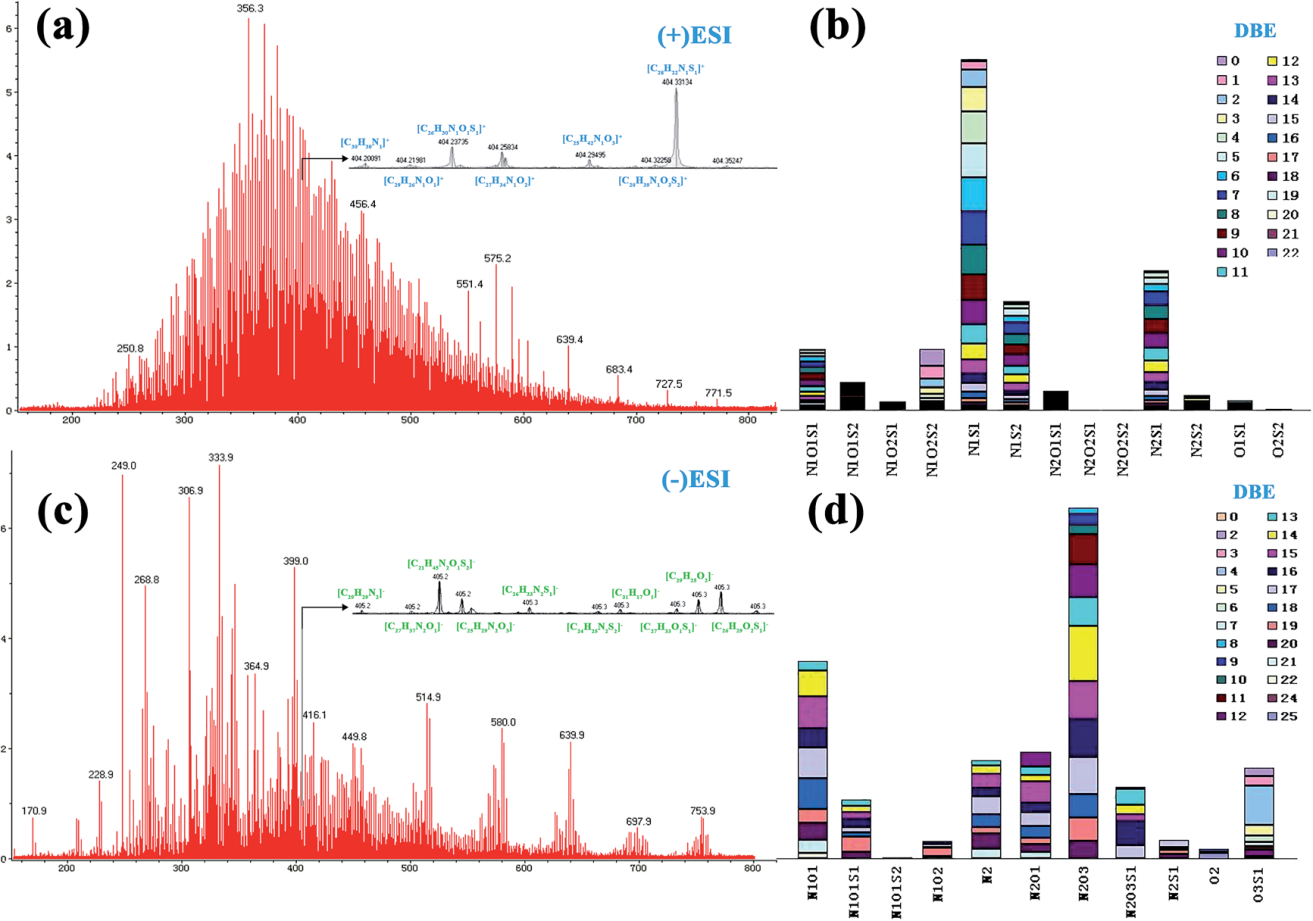
Positive ion ESI FT-ICR mass spectra and at nominal mass, (a); basic nitrogen species and DBE in positive mode (b); negative-ion ESI FT ICR mass spectra and at nominal mass, (c); non-basic/acidic species and their DBE (d).

Fig. 1 shows ESI FT ICR mass spectra analysis for asphaltene sample XJ-1. In the positive mode Fig. 1(a and b) where basic nitrogen compounds where selectively ionized, the mass distribution range (*m*/*z*) was between 100–800 Da. A mono-modal mass distribution was observed to be mainly concentrated in the range 220–780 Da.

In the anion mode, Fig. 1(c and d), majority of the hetero atoms were oxygen-containing species. The mass distribution range was mainly between 170 and 780 Da, and peak intensities were highest between 230 and 420 Da. Considering the mass distribution in both broadband, the average mass of these asphaltene molecules is ∼400 Da. This determination is consistent with what has been reported in earlier studies for asphaltenes molecular weights.^33,34^ Thus an implication that the Xinjiang asphaltenes as well have got low molecular weights.

In both modes the spectra were zoomed to nominal mass to provide a detailed analysis for the spectra. In the positive mode, the spectrum was focused to nominal mass *m*/*z* = 404 whereby the baseline resolution exhibited 10 peaks whereas in negative mode Fig. 1(c) at nominal mass *m*/*z* = 405, the baseline resolution was 12 peaks. These are homologous series that are grouped according to their carbon number, degree of unsaturation (DBE) and heteroatom species (N_*n*_O_o_S_s_), Fig. 1(b and d). Positive ESI sensed thirteen nitrogen species with DBEs' ranging between 0–22, Fig. 1(b).

The abundance of the species was in the order: N_1_S_1_ > N_2_S_1_ > N_1_S_2_ > N_1_O_1_S_1_ ≈ N_1_O_2_S_2_ > N_1_O_1_S_2_ > N_2_O_1_S_1_ > N_2_S_2_ > N_1_O_2_S_1_ ≈ O_1_S_1_ > O_2_S_2_ and majority of the species were in the nitrogen–sulphur classes (N_1_S_1_, N_2_S_1_ and N_1_S_2_). The abundance of N_2_O_2_S_1_ and N_2_O_2_S_2_ was minuscule albeit detectable.

Negative ESI FT ICR MS detected nine (9) non-basic nitrogen compounds and two (2) acidic species in the asphaltenes Fig. 1(d).

The abundance was in the order N_2_O_3_ > N_1_O_1_ > N_2_O_1_ > N_2_ > O_3_S_1_ > N_2_O_3_S_1_ > N_1_O_1_S_1_ > N_2_S_1_ > N_1_O_2_ > O_2_ > N_1_O_1_S_2_ and their DBE were between 0 and 25, majority being nitrogen–oxygen containing species (N_2_O_3_, N_1_O_1_ and N_2_O_1_).

**Fig. 2:**
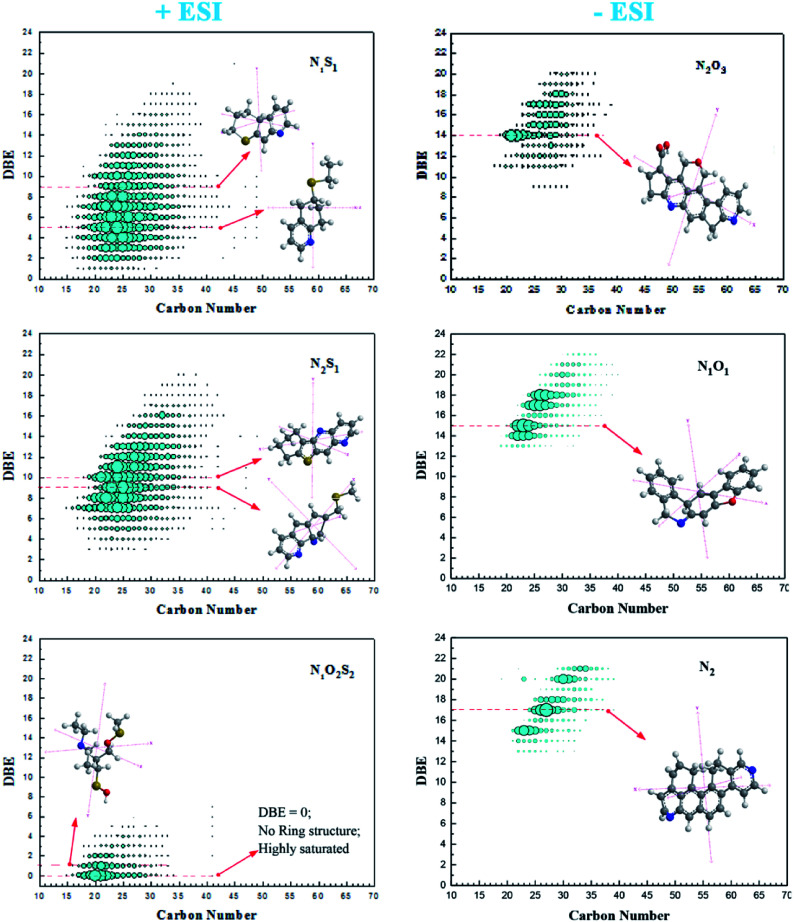
DBE distribution and carbon number for the heteroatom classes detected in positive (right) and negative (left) ESI FT ICR MS.

This high concentration of oxygen species is consistent we the elemental analysis and may be an indicator of some form of biodegradation due to shallowness of the reservoir and somewhat low temperature. The most abundant class had DBEs ranging from 8–14 indicating a high degree of condensation and aromaticity. Compounds with carbon numbers 22–24 and double bond equivalent of 14 were the most abundant class, Fig. 2.

The degree of unsaturation is obvious in Fig. 2 but noticeable observation is the group of compounds with zero DBE that justify existence of typical aliphatic compounds. In this case, its suffice to say, that the basic compounds in the N_1_O_2_S_2_ class identified in positive mode are mostly unsaturated and aliphatic compounds that may be attached to ring systems as branches. Bruno Schuler and his team using AFM measurements showed the possibility of some asphaltene structures being aliphatic in crude oil though with high uncertainty.^9^

The asphaltenes contain heteroatoms that are majorly unsaturated. This signifies presence of various polycyclic aromatic hydrocarbons (PAHs).

### FTIR-analysis of XJ-1 asphaltenes

**Fig. 3:**
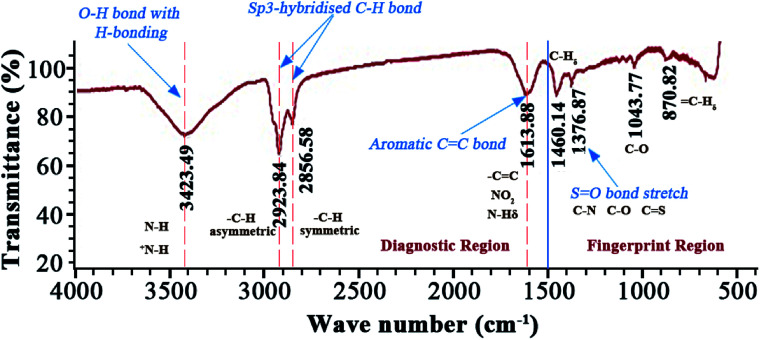
FTIR spectrum of XJ-1 asphaltenes.

The obtained FTIR spectrum of the asphaltenes is presented in the Fig. 3. It has been divided into two important regions: diagnostic (<1500 cm^−1^) and fingerprint (>1500 cm^−1^) region.

It is observed, in the region where wavenumber is greater than 3000 cm^−1^, a broad shaped, medium intensity signal at wavenumber 3423.49 cm^−1^ exists. Signals in this region are attributed to N–H and O–H bond stretching.^31^ However, in this case the broad shape is characteristic of an O–H stretching bond with some hydrogen bonding. This also justifies existence of –OH containing groups like phenols and alcohols. Between 3000 and 2800 cm^−1^, exists sp^3^-hybridized C–H stretching bond. This is an alkyl group and two signals are observed because of the differences in bond stretching. The signal at higher wavenumber (2923.84 cm^−1^) belongs to an asymmetrical bond stretching since it requires a significantly high amount of energy as compared to symmetrical bonds (2856.58 cm^−1^). Between 1700 and 1500 cm^−1^, exists a double bond region of a C=C stretching bond representing aromaticity in the asphaltenes and unsaturation.^32^ Signals from aromatic amines in this region are overlapped by the vibrations from alkyl aliphatic C–H groups. Vibrations from sulfoxide and C–O groups are noticeable in the finger print region. The C–O peak is attributed to stretching in ethers and C–O–H groups in phenols and probably alcohol groups as well.

These deductions emphasize the observations of the ESI FT ICR MS. The absence of the carbonyl peak at ∼1700 cm^−1^ indicates that the oxygen is from alcohols and ether functional groups (–COOR or C–OH groups). It's imperative to note that sulphur is from sulphides, sulfoxides and thiophenes. Even though some of these signals are weak in the FTIR, it cannot be neglected to mention that earlier asphaltene studies^35,36^ with X-ray absorption near edge structure (XANES) have been efficient in identifying these groups.

### ^1^H-nuclear magnetic resonance (^1^H-NMR) analysis

**Fig. 4:**
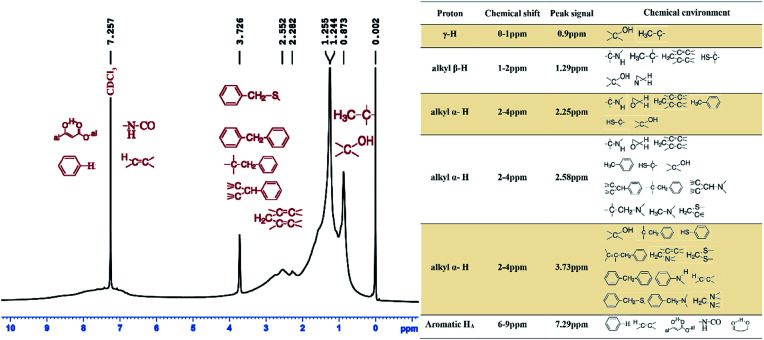
The ^1^H-NMR spectra of XJ-1 asphaltenes.

The obtained ^1^H-NMR spectrum of the asphaltenes is presented in Fig. 4.

Using hydrogen intensities obtained from the spectrum data, elemental analysis and molecular weight; average structural parameters were calculated and shown in Table 3. It is to be noted that since porphyrins have been reported as a main constituent of asphaltenes,^37^ their resonance occurs in the negative region of the spectrum and as such in this analysis, their existence is simply supported by the presence of Ni/*V* metallic elements. Chemical shifts in the regions 6–9 ppm, 2–4 ppm, 1–2 ppm and 1–0 ppm are of significant value since the aromatic, alpha, beta and gamma alkyl hydrogens respectively resonate in those regions.

**Table 3:** Average structural parameters of different asphaltenes

Structural parameters	
*w* (C) %	75.61
*w* (H) %	6.35
H_AU_/C_A_	0.41
Avg. number of aromatic ring carbon per molecule, C_A_	66.20
Avg. number of naphthenic ring carbon per molecule, C_N_	26.00
Avg. number of alkyl carbon number, C_P_	35.91
Avg. number of aromatic rings per molecule, R_A_	22.65
Avg. number of naphthenic rings per average molecule, R_N_	8.11
Ratio of aromatic carbon to total carbon, *f*_a_	0.48
Ratio of alky carbon attached to naphthalene rings, *f*_p_	0.27
Hydrogen–carbon ratio, H/C	1.37

A chemical shift at 0.9 ppm, is from hydrogen of a –CH_3_ group belonging to either an iso-paraffin or normal alkyl paraffin^38,39^ and further away from the aromatic ring. Resonance at 1.29 ppm, is most probably from CH_2_- at beta position from the aromatic ring in a long alkyl aliphatic branch since resonance in this group has been reported to be 1.30 ppm which is very close to 1.29 ppm.^39,40^ This explains the zero DBE observed in ESI FT ICR mass spectra. However, because we are dealing with a complex molecule, it is safe to deduce that the proton may as well be from a β-positioned = CH- in an alkyl branch.^40^ Resonance signals at 2.25 ppm and 2.58 ppm are most probably from a –CH_3_ (methyl group) with the carbon atom at α-position in respect to the aromatic carbon to which it is attached.

Literature sources report signals from this methyl group at 2.30 ppm and 2.60 ppm.^39,41^ It is also to be noted that α-protons in aromatic rings on =CH- and CH_2_- groups as well resonate between 2.4–3.5 ppm.^39–41^ The proton shifts at 3.72 ppm is attributed to the bonding –CH_2_- group in the diphenyl chemical environment, Fig. 4.

The signal at 7.29 ppm is normally attributed to the deuterated chloroform but it should be noted that due some overlapping issues, some chemical shifts that exist in this range may exist. Among them that have been documented in earlier studies include the di-, tri- and tetra-aromatic ring groups.^38,40,42^ The proton in these groups resonates at concentrations between 7.2–8.3 ppm.

On average more γ hydrogens were protonated implying a vast network of alkyl branches longer than ethyl groups attached to the ring network. This is also evinced in the average chain length, *n*, of 4.37.

As per the calculated structural parameters, Table 3, the asphaltenes, contain as up to 66.20 aromatic ring carbons and an aromaticity of about 48% that results into formation of a conjugated π bond system. Besides, a low H_AU_/C_A_ ratio was observed that represents high tendency of aromatization, condensation of the rings thus easier formation of conjugated π bond system. However, it should be noted that when the degree of alkylation increases, the spatial steric hindrance between asphaltene molecules also increases, hindering association between molecules hence reducing the number of structural units and consequently the low molecular weights observed in ESI FT ICR spectra.

**Fig. 5:**
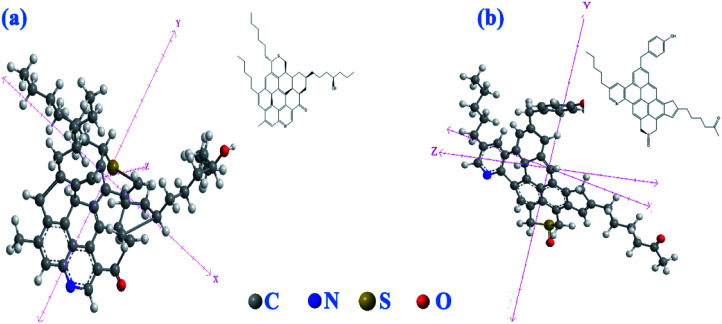
Assigned structure for the Xinjiang asphaltenes, (a) island like model, (b) aryl linked like structure.

It is also plausible to deduce that the conjugated π bond system causes polarizability in the molecules which creates a series of both deficient and electron rich molecules that counters the hydrogen bonding in the asphaltenes as seen in solubility studies for asphaltenes.^43–45^ Based on these deductions the asphaltenes may take on the structures as observed in Fig. 5(a and b).

The predicated structures are similar to those reported for crude oil asphaltenes using STM/AFM results^9^ and considering the observed polycyclic aromatic system with side branches, Fig. 5(b), this structural assignment is consistent with the aryl linked core model Fig. 5(b) due to the probable diphenyl group in the NMR spectrum albeit the island model is still plausible Fig. 5(a).

## Conclusions

In this study, with hindsight we have been able to analyse Xinjiang asphaltenes using NMR, ESI FT ICR MS, and FTIR to characterize their internal makeup.

We conclude that generally, they have low metallic content, a modest molecular weight, high degree of unsaturation and contain homologous series with long alkyl branches. The functional groups of the homologous series are alcohols, ethers, sulphides, amides and sulfoxides.

The inferences are based entirely on spectroscopy information’ but the asphaltene molecules are more complex and also contain Ni/*V* metallic constituents. While we couldn't successfully ascertain the real structure of the asphaltenes due lack of STM/AFM measurements we were able to develop a modest thought of their internal likeness based on scientific information collected from high resolution spectroscopy. The structural assignments for these asphaltenes are consistent with the “island” and “aryl-linked core” models.

In our next phase, we will focus on these findings to develop an environmentally friendly inhibitor that remedies the underlying problems posed by asphaltenes during oil production.

## Experiment and methods

### Asphaltene extraction

Using the Polar separation method, the main components/fractions (saturates, aromatics, resins, asphaltenes) in the heavy oil were separated. The separation was in accordance with the ASTM-2007-03 standard test procedure.^5^

### Fourier transform-infrared spectroscopy (FT-IR)

Conducted at room temperature; the MAGNA-IR 560 ESP spectrometer with a scan range between 4000–400 cm^−1^ was used to analyse the asphaltene sample at resolution of 0.35 cm^−1^. The spectrometer has a 30 000 : 1 signal to noise ratio. The data was processed and output using the OMNIC software.

### NMR spectroscopy analysis

The experiment was performed at 24.5 °C using deuterated chloroform (chloroform-d) as the solvent and tetramethylsilane (TMS) as the internal calibration standard.

The Avance-600 MHz nuclear magnetic resonance spectrometer was used in this investigation.^25,46^

### ESI FT-ICR MS analysis

The asphaltenes' solution was prepared in reference to Klein's experiment^47^ and then infused into Bruker Apex-Ultra FT-ICR MS fitted with a 9.4-Tesla super-conducting magnet and an ESI source using an Apollo electrospray at a rate of 180 μL h^−1^. The ESI was operated both in positive/negative modes under voltages −4/3.5 kV emitter voltage, −4.5/4 kV capillary front and −320/−320 V capillary end voltage. Detailed experimental procedure and data processing is reported elsewhere in earlier academic advances.^21,47–49^

### Metallic and non-metallic analysis

Metallic elements were determined using the PerkinElmer Optima 3000 emission spectrometer that can measure wavelengths between 167–782 nm for over 5000 emissions. The liquid sample was rapidly atomized using an argon plasma at 7000 °C and electrons excited for detection and analysis using WinLab32 software. The non-metallic elements were analysed using the Vario EL Elementar equipment. It was conducted in accordance to the Petrochemical industry standard GB/T 19143-2003.

## Conflicts of interest

There are no conflicts to declare.

## Supplementary Material
